# Adipose tissue macrophages in aging-associated adipose tissue function

**DOI:** 10.1186/s12576-021-00820-2

**Published:** 2021-12-04

**Authors:** Bangchao Lu, Liang Huang, Juan Cao, Lingling Li, Wenhui Wu, Xiaolin Chen, Congzhu Ding

**Affiliations:** Department of Geriatrics, Nanjing Drum Tower Hospital, The Affiliated Hospital of Nanjing University Medical School, Nanjing, Jiangshu China

**Keywords:** Adipose tissue macrophages, Age, Insulin resistance

## Abstract

“Inflammaging” refers to the chronic, low-grade inflammation that characterizes aging. Aging, like obesity, is associated with visceral adiposity and insulin resistance. Adipose tissue macrophages (ATMs) have played a major role in obesity-associated inflammation and insulin resistance. Macrophages are elevated in adipose tissue in aging. However, the changes and also possibly functions of ATMs in aging and aging-related diseases are unclear. In this review, we will summarize recent advances in research on the role of adipose tissue macrophages with aging-associated insulin resistance and discuss their potential therapeutic targets for preventing and treating aging and aging-related diseases.

## Introduction

The world has entered an aging society. In 2015, an estimated 617 million people (8.5%) in the world were age ≥ 65 years and is expected to more than double to ≈1.6 billion (17%) in 2050 [1]. The growing health, economic and social problems brought about by aging have attracted worldwide attention.

Aging is a process in which the body gradually loses its physiological integrity and organ function is damaged, leading to death. Aging is always accompanied by obesity and metabolic dysfunction, including insulin resistance. Recent studies have shown that aging-associated insulin resistance is related to immunosenescence and inflammaging [2–4]. ‘Inflammaging’ refers to the chronic, low-grade inflammation that characterizes aging. Inflammaging is a complex balance between pro- and anti-inflammatory responses, which is centered on macrophage and involves multiple tissues and organs, including adipose tissue.

Aging is also associated with important changes in the innate immune system. Macrophages perform important innate immune functions including phagocytic clearance of dying cells. The polarized state of macrophages can be classified into two subsets: classically activated (M1) and alternatively activated (M2). M1 macrophages highly express genes related to pro-inflammatory cytokines or oxidative stress, including genes related to TNF-alpha, IL-6, MCP-1, and iNOS, whereas M2 macrophages highly express other genes that encode arginase-1, Mrc1(CD206), Ym1, and IL-10, an anti-inflammatory cytokine [5].

In obesity, it is well known that adipose tissue macrophages (ATMs) play a key role in obesity-associated insulin resistance. A large number of macrophages in the blood are collected into adipose tissue [6]. The number of ATMs increased from 5 to 50% of the total number of adipose tissue cells and transformed from M2 to M1 cells [7]. Many M1 ATMs surround the necrotic adipocytes to form crown-like structures (CLS), a hallmark of low-grade inflammation and insulin resistance [8, 9].

There are similarities and differences between inflammation in adipose tissue caused by aging and that caused by obesity. So far, few studies have focused on the effects of ATM on aging. This article will describe the change of adipose tissue macrophages and its role on aging-associated insulin resistance.

### Adipose tissue inflammation in obesity and aging

Adipose tissue (AT) is the largest endocrine organ in human. Adipose tissue is composed of a variety of cells, including adipocytes, pre-adipocytes, endothelial cells, fibroblasts, nerve cells, macrophages and lymphocytes. The number and phenotypes of these cells are dynamic in obesity and aging [10].

As we age, many changes have taken place in adipose tissue:

1. Fat mass tends to be preferentially distributed in the abdominal region. Compared with subcutaneous fat, visceral fat increased more significantly.

2. Ectopic lipid accumulation occurs not only in visceral depots, but also in bone marrow or muscle, among other tissues [11].

3. There are an increasing number of senescent cells in the adipose tissue.

4. Aberrant secretion of adipokines.

5. The aged adipose tissue is also characterized by reduced adipocyte size, tissue fibrosis, endothelial dysfunction, and reduced vascularization and angiogenic capacity [12].

Alterations in adipose tissue are major contributors to age-related metabolic dysfunctions and longevity [13–15]. There are fundamental cellular and molecular differences in adipose tissue inflammation between diet-induced obesity and age-associated obesity [15].

Obesity-related AT inflammation is initiated by the inability of adipose tissue to buffer dietary lipids [16]. Excessive lipid causes adipocyte stress lipotoxicity in the liver and skeletal muscle increases reactive oxygen species and activates serine threonine kinases, such as c-jun N-terminal kinase (JNK), IκB kinase (IKK), and protein kinase C (PKC). These events disrupt insulin receptor signaling cascades and promote insulin resistance [17]. Adipocyte death in obese humans will increase the stress of ATMs, leading to inflammation activation. A large number of ATMs infiltrate AT and secrete inflammatory factors. In addition, as adipose tissue is an endocrine organ, adipocytes also can secrete adipokines. During obesity, adipocytes increase pro-inflammatory cytokine and chemokine secretion of IL-6 and TNF-alpha, where TNF-alpha has been shown to increase insulin resistance in diet-induced obese mice [18].

In Lumang’s study [19], young and old mice adipose tissue were divided into ATMs〔CD11b( +)], adipose tissue stromal cells (ATSCs) [CD11b (−)) and adipocytes. The cytokines and chemokines produced by these cells were analyzed, respectively. Although these three components secrete IL-6 and MCP-1, in vitro, the levels of ATSCs and ATMs were significantly higher, implicating that the main contributors of adipose tissue inflammation during aging were the resident fat immune cells rather than adipocytes.

In addition, senescent cells in adipose tissue become a potential source of aging-associated AT inflammation. Recent studies have shown that visceral and groin adipose tissue in mice contains large amounts of p16 (Ink 4 a)-positive senescent cells [10]. These senescent cells secrete various types of pro-inflammatory cytokines, such as IL-6, IL-8 and TNF-alpha, known collectively as the aging-related secreted phenotype (SASP) [20]. Because the old immune system is not effective enough in removing senescent cells [21, 22], these pro-inflammatory factors gradually accumulate with aging.

Adipose tissue inflammation is closely related to innate adipose immune cells during aging. The main immune cell types in adipose tissue include macrophages (ATMs) and T lymphocytes, and other immune cell types [23, 24]. In this review, we focus on changes and its mechanisms of ATMs in aging.

## Adipose tissue macrophages in aging

Adipose tissue is an immunometabolically active organ in a constant state of flux [25]. The metabolic function of adipose tissue changes with increasing age. Aged adipose tissue becomes less sensitive to insulin, lipolytic stimulation and fatty acids [13]. In animal models, inflammation and metabolic regulation of adipose tissue affect animal life span, suggesting that adipose tissue, especially white adipose tissue, now emerges as a pivotal organ controlling lifespan [26, 27].

Macrophages are the inflammatory source in adipose tissue. These innate immunity cells are derived from bone marrow hematopoietic stem cells [28]. The number of macrophages in adipose tissue depends on the proliferation of macrophages in local adipose tissue and the recruitment of monocytes in blood circulation to adipose tissue [29].

Adipose tissue macrophages (ATMs) are the greatest proportion of leukocytes in adipose tissue. ATMs were classified under the prototypical dichotomy of M1 “classically” activated macrophages (CD11c + CD206 −) and M2 “alternatively” activated macrophages (CD11c − CD206 +). M1 macrophages have high phagocytic and bactericidal potential, secrete pro-inflammatory cytokines and activate Th1. M2 macrophages interact with Th2 lymphocytes to promote anti-parasitic activity, wound healing and tissue repair as well as produce anti-inflammatory cytokines that prevent excessive immune responses. Lumeng et al. [19] reported an inflammatory double-negative ATMs (CD11c − CD206 − , DN) in mice. However, this kind of ATM phenotype has not been observed in humans.

In young adipose tissue, most of ATMs are M2 cells. Their functions are not only anti-inflammatory, but also efferocytosis, lipid buffering, angiogenesis, regulation of iron flux. They can help maintain homeostasis of AT. Aging alters the balance of visceral adipose tissue macrophages toward the pro-inflammatory M1 phenotype and DN ATMs [19]. It is likely not only the increase in inflammatory adipose tissue macrophages, but also the decreased homeostatic function of the resident M2 cells during aging.

The phagocytic function and antigen presenting ability of adipose tissue macrophages decreased with age [30]. Proinflammatory cytokines such as interleukin (IL-6), tumor necrosis factor α (TNF-alpha) and IL-1β secreted by M1 macrophages are elevated both in aged mice [19, 31, 32] and older humans [33]. These cytokines can interfere with insulin receptor signaling pathways, which in turn induce local (fat) and systemic (liver and skeletal muscle) insulin resistance. M1 macrophages are engaged in early stage of aging and age-related metabolic syndrome such as atherosclerosis, obesity, type II diabetes.

**Fig. 1:**
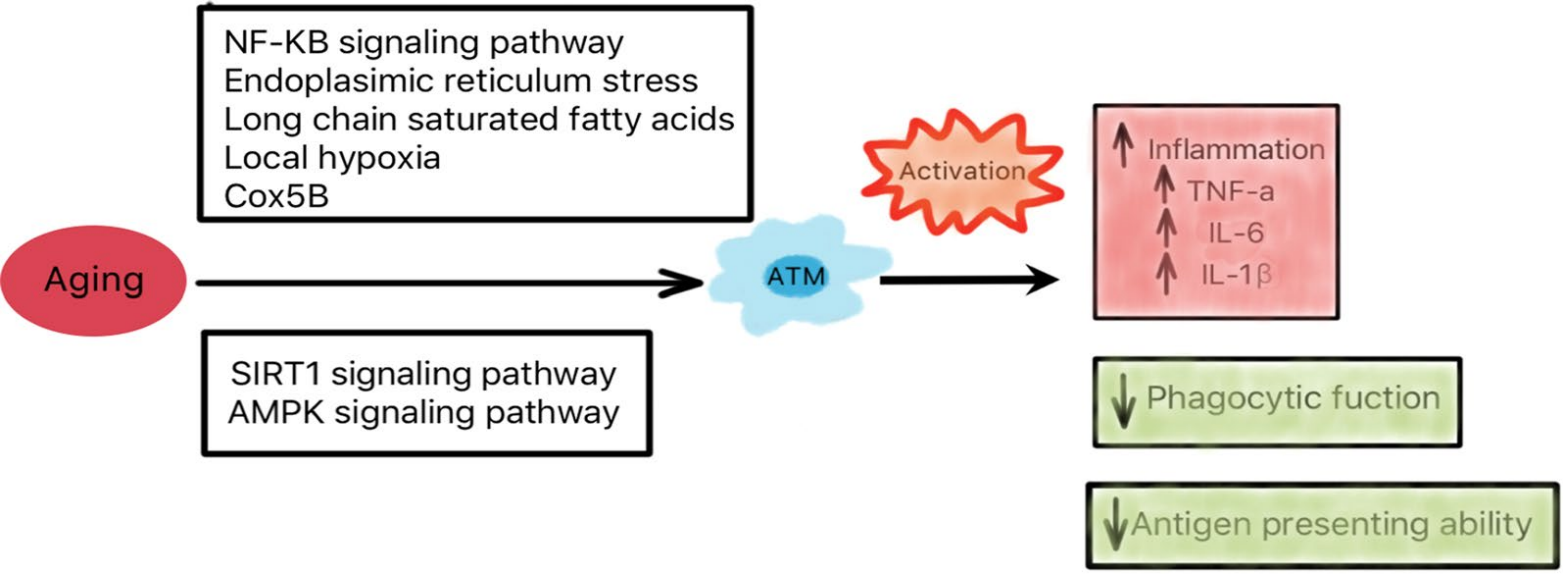
Macrophages in young, obese and old adipose tissue. In young adipose tissue, most of ATMs are M2 cells. Their functions are not only anti-inflammatory, but also efferocytosis, lipid buffering, angiogenesis, regulation of iron flux. They can help maintain homeostasis of adipose tissue (AT). In obesity, a large number of macrophages in the blood are collected into adipose tissue. The number of ATMs increased from 5 to 50% of the total number of adipose tissue cells and transformed from M2 to M1 cells. Many M1 ATMs surround the necrotic adipocytes to form crown-like structures (CLS), a hallmark of low-grade inflammation and insulin resistance. In aging adipose tissue, the total ATM content is unchanged, but the ratio of M1/M2 ATMs is increasing. Aging alters the balance of adipose tissue macrophages toward the pro-inflammatory M1 phenotype

In mice, Lumeng [19] found that total ATM content is unchanged in old mice by observing young (3–4 months) and old (18–22 months) C57BL/6 mice. With age, the ratio of pro-inflammatory M1 ATMs to resident M2 ATMs was significantly increased. A decrease in PPARγ expression in ATMs is associated with this change. CCR2-dependent chemokine pathway may also be related to ATM recruitment in age. In addition, non-macrophage stromal cells and adipocytes of old mice can activate macrophages through paracrine effect (Fig. 1).

In humans, there are few studies on human adipose tissue macrophages due to the difficulty of sampling. Victoria [34] observed healthy Pima Indians, found that subcutaneous adipose tissue macrophage content increases steadily with age, independent of sex, ethnicity, adipose tissue depot, and diagnosis of diabetes. At about 30 years of age, the number of ATMs in human will reach a peak and then drop slightly until 45 years. Consistent with studies in mice, adipose tissue macrophage content (ATMc) per se is not a direct cause of insulin resistance. The expression of the macrophage activation marker PAI-1 was associated with insulin resistance, suggesting that macrophage activation, rather than number, may be more important in mediating the association between inflammation and lower insulin-mediated glucose uptake.

## Aged ATMs in lipolysis

Aging is a chronic and complex physiological process, which gradually worsens the energy homeostasis [35]. The dynamic balance of lipid storage and lipolysis in aged adipose tissue is not well controlled. Mobilization of FFAs is dysregulated, causing visceral adiposity, lower exercise capacity, and cold intolerance. Adipose macrophages are involved in age-related reduction of lipolytic activity. Camell et al. [36] showed that aged ATMs decrease catecholamine-dependent lipolysis in a NLRP3 inflammasome-dependent manner in mice. Deletion of NLRP3 in aging restored catecholamine-induced lipolysis through downregulation of growth differentiation factor-3 (GDF3) and monoamine oxidase-a (MAOA) that is known to degrade norepinephrine (NE). Inhibition of MAOA in macrophages restored lipolysis by increasing key lipolytic enzymes. However, in human adipose tissue, MAOA is mainly expressed in mature adipocytes. Gao et al. [37] report that age-Induced reduction in human lipolysis is related to catecholamine pathway in subcutaneous adipocytes, suggesting species-specific differences in aging mechanisms.

## Mechanisms for regulation of functions of ATMs

Aging is mainly associated with adipose tissue macrophage polarization, rather than ATMs recruitment and infiltration like obesity. However, the molecular mechanisms responsible for ATM activation and polarization remain unknown. Here are several signaling pathways related to it, so far.

### NF-ΚB

NF-κB is considered to be a central transcription factor in the regulation of inflammatory response, because it controls the synthesis of most inflammatory markers and mediators (including TNF-alpha, IL-6, IL-1, IL-8, MCP-1, iNOS, COX-2 and adhesion molecules) [38], whereas PPAR-γ antagonizes NF-ΚB-associated activation.

Wu [31] found that the mRNA expressions of pro-inflammatory cytokines IL-1, IL-6, TNF-alpha and COX-2 in visceral adipose tissue of aged C57BL mice were significantly higher than those of young mice, while the expression of anti-inflammatory PPAR-γ was lower than that of young mice. When peritoneal macrophages were cultured with the extract of aged mice adipocytes, it was found that they were easy to polarize to M1, which can produce more TNF-alpha and IL-6. He also found that sphingolipid ceramide was higher in old compared with young adipose tissue. Ceramide is involved in age-associated up-regulation of IL-6 and TNF-alpha. Reducing ceramide levels or inhibiting NF-ΚB activation decreased those cytokine production, whereas the addition of ceramide has the opposite effect, suggesting that ceramide-induced activation of NF-ΚB plays a key role in adipose tissue inflammation. These observations in mice likely correspond to those made in obese adipose tissue from humans.

### Endoplasmic reticulum stress

In obesity, endoplasmic reticulum stress is involved in adipose tissue inflammation and insulin resistance. Endoplasmic reticulum stress can inhibit insulin signaling by activating c-Jun N-terminal kinase and serine phosphorylation of IRS-1 [39].

In recent years, Ghosh AK [40] reported ER stress markers are also elevated in aging ATMs. Old ATMs are relatively more sensitive to ER stress compared to young ATMs. Endoplasmic reticulum stress inhibitors can inhibit the production of TNF-alpha by ATMs in old mice. Autophagy is programmed cell survival. But too much or too little autophagy can damage cells. Abnormal autophagy in aging adipose tissue can increase endoplasmic reticulum stress and local inflammation [41].

### SIRT1

Sirtuins are a group of NAD+-dependent protein deacetylases, which are considered to be the key regulators of natural aging and other stress-related diseases [42, 43]. Among them, SIRT1 is an important factor involved in adipocyte metabolism. In mature adipocytes, SIRT1 promotes fat mobilization through repression of PPARγ [44] and protects cells from TNF-alpha-induced insulin resistance [45]. So, in obesity-related insulin resistance of mice, Hui et al. [46] found that SIRT1 in adipocytes is more critical than SIRT1 in other parts. Because SIRT signal in adipocytes is involved in regulating the expression and secretion of some adipokines, such as adiponectin, MCP-1 and interleukin 4. SIRT knockout of rat adipocytes will accelerate the recruitment of macrophages to adipose tissue and polarize into M1 type. In human subjects, SIRT1 level is inversely related to BMI and adipose tissue macrophage infiltration. Overexpression of SIRT1 effectively blunts obesity-induced adipose tissue macrophage infiltration [47].

SIRT1 activator can improve the health status and prolong the life span of mice [48, 49]. Aging can reduce NAD + and SIRT1 activity [50], while energy restriction and exercise can stimulate SIRT1 activity. Aging can reduce the activity of SIRT in hypothalamus, promote leptin resistance and increase obesity [51, 52]. However, the role of SIRT1 on ATMs activation and polarization in aging is unknown. There are few related reports, which are worthy of further study.

### AMPK

AMPK is a central regulator of fatty acid, cholesterol, and glucose homeostasis through phosphorylation of metabolism-regulating enzymes including acetyl-CoA carboxylase (ACC), glycogen synthase (GS), glucose transporter 4 (GLUT4), HMG-CoA reductase, hormone-sensitive lipase (HSL), and mammalian target of rapamycin (mTOR) [53].

As a potent counter-regulator of inflammatory signaling pathways, AMPK can inhibit pro-inflammatory responses in macrophages and promote macrophage polarization into anti-inflammatory phenotype. Anti-inflammatory cytokines (i.e., IL-10 and TGFβ) can induce the rapid phosphorylation/activation of AMPK in macrophages, whereas pro-inflammatory stimulus (LPS) resulted in AMPK dephosphorylation/inactivation. AMPKalpha1 is the main AMPKalpha isoform of macrophages. Inhibition of AMPKalpha expression increased LPS-induced macrophage inflammatory cytokine production, however, expression of a constitutively active AMPKalpha1 results in the opposite consequences. In addition, AMPK was found to reduce LPS-induced IκB-alpha degradation and enhanced Akt activation, accompanied by inhibition of GSK3-β and activation of CREB. Therefore, it is speculated that AMPK is the master switch of macrophage polarization [54].

AMPK is a pro-longevity kinase [55]. AMPK activity in tissues decreased gradually during aging. Recent studies have shown that activating AMPK is sufficient to regulate longevity and extend calorie restriction-induced lifespan in many organisms. Salminen et al. [56] reported several key pro-longevity pathways regulated by AMPK, including inhibition of CRTC-1/CREB and NF-κB and mTORC1, as well as activation of SIRT1, Nrf2, FOXO1 and ULK1. There is a low level of inflammation in the aging process. AMPK can reduce the inflammatory responses by inhibiting NF-κB signaling. AMPK does not directly phosphorylate NF-κB subunits, but acts on NF-κB by regulating downstream factors, such as SIRT1, PGC-1α, p53, and Forkhead box O (FoxO) factors [57].

It is known that AMPK is an important inflammatory suppressor, and AMPK regulates macrophage polarization in obese adipose tissue inflammation [58, 59]. The M1–M2 polarization of macrophages is regulated by AMPK. After the AMPK β1 subunit gene was knocked out, the activity of AMPK, the phosphorylation of acetyl-CoA carboxylase and the content of mitochondria in macrophages decreased, resulting in the decrease of fatty acid oxidation rate. β1(−/−)macrophages showed increased levels of diacylglycerol and inflammatory markers. AMPKalpha1 knockdown macrophages could express and secrete more TNF-alpha and IL-6 under the stimulation of lipopolysaccharide (LPS) [54]. In addition, AMPK-specific activator inhibited LPS-induced TNF-alpha expression in mouse macrophages. In the visceral adipose tissue of obese people, the decrease of AMPK activity is closely related to adipose tissue inflammation [60]. AMPK also can enhance SIRT1 by increasing NAD/NADH ratio and decreases adipose tissue macrophage infiltration and inflammation [61, 62].

To date, the role of ATMs AMPK signal in aging is unclear. This aspect is worthy of further study.

### Long-chain saturated fatty acids

In obesity, saturated fatty acid palmitate can increase the retention of macrophages by increasing the expression of netrin-1 in obese adipose tissue, leading to insulin resistance [63]. Long-chain saturated fatty acids, rather than unsaturated fatty acids, induce macrophages to produce inflammatory response through JNK signaling pathway [64].

. In addition to ectopic fat deposition, the level of free fatty acids in the elderly is increased. Increased free fatty acids, especially saturated fatty acids, may be the drivers of insulin resistance and inflammation in the elderly. Ghosh [65] found that free fatty acid (FFA)-induced aging adipose tissue inflammation and insulin resistance are dependent on the TLR4 signaling.

### Others

#### Local hypoxia

The aged adipose tissue is also characterized by reduced adipocyte size, tissue fibrosis, endothelial dysfunction, and reduced vascularization and angiogenic capacity [12]. Local hypoxia of adipose tissue may cause the accumulation and inflammatory polarization of ATMs [66, 67].

#### COX5B

Mitochondrial cytochrome oxidase subunit 5B (cox5b) can induce the production of HIF-1α. Studies have found that aging can reduce the content of cox5b in adipose tissue [68]. With age, the decrease of Cox 5B in human visceral adipose tissue not only increases HIF-1α, but also increases the storage of lipid in cells, which promotes the expansion of adipocytes. If this hypertrophic expansion continues, the stress signals that promote macrophage infiltration will be released, as observed in obesity [69, 70].

## Conclusions and perspectives

**Fig. 2:**
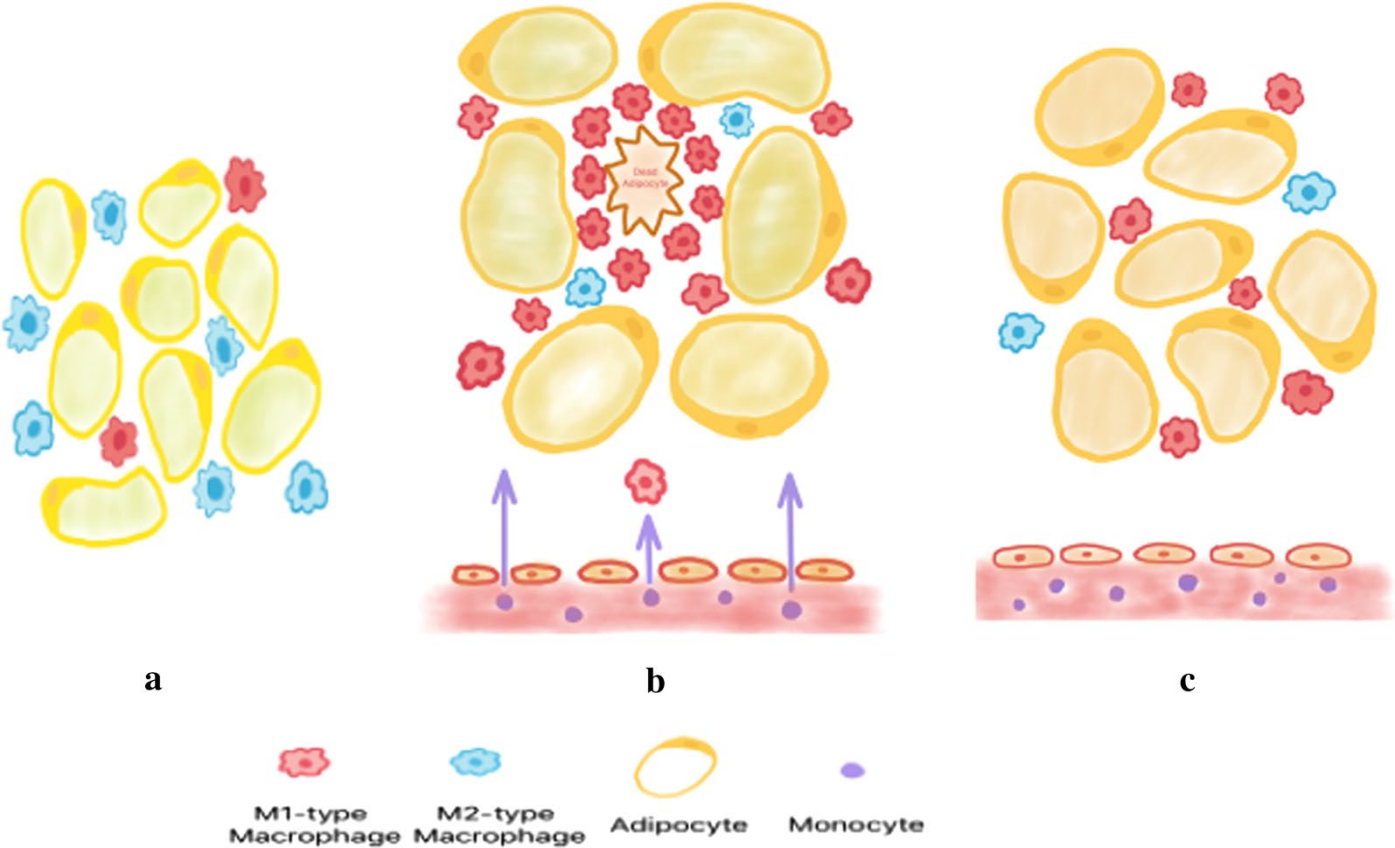
Effect of aging on adipose tissue macrophages

Aging is commonly associated with low‐grade adipose inflammation, which is closely linked to insulin resistance. ATMs play an important role in adipose tissue inflammation; limited reports have also shown about ATM in aging. The ratio of M1/M2 ATMs is increasing, not the total ATM content in aging. The polarization mechanism of ATMs and its role in aging-related metabolic dysfunction are mainly related to factors such as NF-κB, endoplasmic reticulum, long-chain saturated fatty acids and hypoxia. Several signaling pathways, especially AMPK and SIRT1, need to be further elucidated in ATMs activation (Fig. 2).

The following questions will be the focus of further interest and investigation in the coming years:

1. Which inflammatory pathways are most relevant to ATMs in aging?
2. How do macrophages interact with other immune cells in the AT?

Perhaps most importantly, how can ATMs be modulated to protect against the metabolic effects of aging.

## Data Availability

Not applicable.
